# Not Your Typical V-Tach: Cocaine’s Cardiac Trickery

**DOI:** 10.51894/001c.144449

**Published:** 2025-09-30

**Authors:** Kamel Faraj

**Affiliations:** 1 Department of Emergency Medicine Henry Ford Wyandotte

## 76

### INTRODUCTION

Cocaine, the second most widely used illicit drug in the U.S., affects the central nervous and cardiovascular systems by inhibiting the reuptake of norepinephrine and epinephrine. This potent sympathomimetic drug can lead to life-threatening complications, including cardiac arrest or respiratory failure, particularly due to its cardiotoxic effects, such as sodium channel blockade and the resultant QRS widening. This case highlights a patient who developed new-onset seizures and wide-complex tachycardia after heavy cocaine use.

### CASE REPORT

A 44-year-old male with no significant medical history was found unresponsive by his wife, exhibiting seizure-like activity. EMS reported ongoing seizures and a concerning rhythm resembling ventricular tachycardia. Upon arrival, the patient was actively seizing and was treated with lorazepam, resolving the seizure. His Glasgow Coma Scale (GCS) score was 6, and he was intubated. Telemetry revealed multiple runs of wide-complex tachycardia (120–140 bpm) with an initial QRS of 166 ms.

Despite the wife denying illicit drug use or possibility of overdose, tricyclic antidepressant (TCA) toxicity was suspected. The patient was treated with two amps of sodium bicarbonate, which led to resolution of the tachycardia and narrowing of the QRS to 116 ms. Hours later, the patient’s friend disclosed heavy cocaine use the night before. The patient was extubated the following day and discharged 48 hours later at his baseline.

### DISCUSSION/CONCLUSIONS

This case emphasizes the importance of a broad differential diagnosis for young patients with wide-complex tachycardia and seizures, especially in the absence of a clear cardiac history. While wide-complex tachycardia with a QRS >160 ms is often assumed to be ventricular tachycardia, it’s crucial to consider other causes like toxin-induced sodium channel blockade. Cocaine-induced sodium channel blockade exacerbates QRS widening, and sodium bicarbonate treatment can counteract this effect, potentially preventing a fatal outcome. If standard ACLS protocols with sodium channel blockers had been used, the patient could have deteriorated.

This case underscores the need for emergency physicians to maintain a high index of suspicion for cocaine toxicity, even when family members deny drug use. Early recognition and prompt treatment are critical to prevent catastrophic outcomes.

